# Supplementary material for: Influence of ionic conditions on knotting in a coarse-grained model for DNA
Source: Front Chem. 2023 Jan 17;10:1096014. doi: 10.3389/fchem.2022.1096014 (PMC9887150; doi:10.3389/fchem.2022.1096014)
Supplement: Supplementary file 1 [file DataSheet1.pdf]

SUPPLEMENTARY INFORMATION

Influence of ionic strength on persistence length and knotting probability

In our simulations, we have assumed a persistence length of $50\ nm$ over the entire range of ionic conditions. However, experimental studies (Sobel and Harpst, 1991; Kam et al., 1981; Manning, 1981; Post, 1983), computer simulations (Rieger and Virnau, 2018; Savelyev, 2012) and theoretical works (Manning, 2006) indicate that for short strands (in the kilo base regime) persistence length decreases considerably (to about 30 to 35 nm) in high salt conditions and also depends somewhat on the actual ions used in the buffer.

In this section, we argue that our simplified approach is nevertheless justified at least with respect to knotting probabilities. Note, that in terms of our coarse-grained model, a reduction of persistence length effectively corresponds to a decrease in stiffness according to equation 2 from the main text. To test this effect, we have rescaled our simulations for physiological conditions ($\kappa = 11.673$ and $d = 4.465\ nm$ corresponding to $l_p = 50\ nm$) to 1M ($\kappa = 11.673$ and $d = 2.95\ nm$ corresponding to $l_p = 34.4\ nm$, compare with eq. 2) and compared those results with our simulations for 1M assuming a persistence length $l_p = 50\ nm$ (at $\kappa = 16.949$ and $d = 2.95\ nm$). As shown in Fig. 1 knotting probabilities are only affected marginally as they depend little on stiffness in this regime as already noted, e.g., by Virnau et. al. in (Virnau et al., 2013).

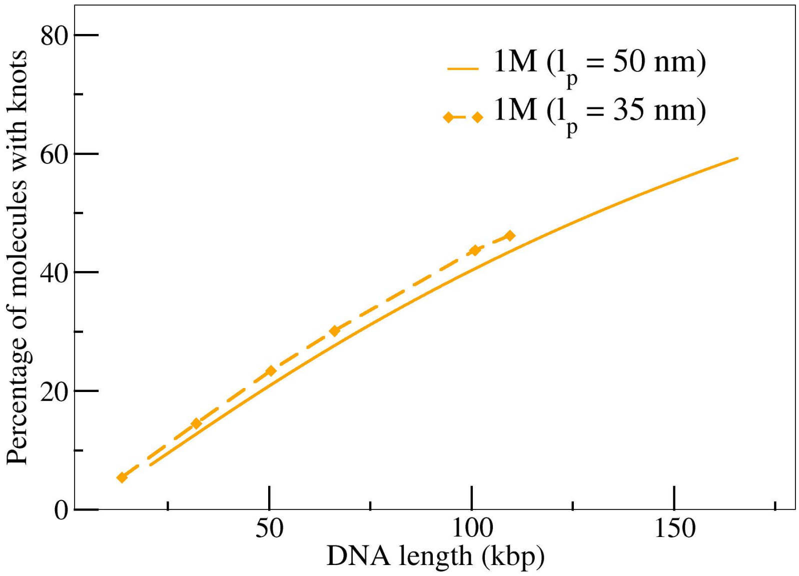

Figure 1. Percentage of knotted strands as a function of DNA length at 1M salt concentration assuming a persistence length l_p of 50 or 35 nm.

Furthermore, Brunet et. al (Brunet et al., 2015)) showed that in high salt conditions persistence length also increases with DNA length, so the effective persistence length might actually be closer to $50\ nm$ for strand sizes considered in our study. This, together with the fact that knotting probability only changes little with stiffness for large values of κ (Fig. 1) justifies our simplified assumption.

REFERENCES

Brunet, A., Tardin, C., Salomé, L., Rousseau, P., Destainville, N., and Manghi, M. (2015). Dependence of DNA persistence length on ionic strength of solutions with monovalent and divalent salts: A joint theory–experiment study. *Macromolecules* 48, 3641–3652. doi:10.1021/acs.macromol.5b00735

Kam, Z., Borochov, N., and Eisenberg, H. (1981). Dependence of laser light scattering of DNA on NaCl concentration. *Biopolymers* 20, 2671–2690. doi:https://doi.org/10.1002/bip.1981.360201213

Manning, G. S. (1981). A procedure for extracting persistence lengths from light-scattering data on intermediate molecular weight DNA. *Biopolymers* 20, 1751–1755. doi:https://doi.org/10.1002/bip.1981.360200815

Manning, G. S. (2006). The persistence length of DNA is reached from the persistence length of its null isomer through an internal electrostatic stretching force. *Biophysical Journal* 91, 3607–3616. doi:https://doi.org/10.1529/biophysj.106.089029

Post, C. B. (1983). Excluded volume of an intermediate-molecular-weight DNA. a Monte Carlo analysis. *Biopolymers* 22, 1087–1096. doi:https://doi.org/10.1002/bip.360220406

Rieger, F. C. and Virnau, P. (2018). Coarse-grained models of double-stranded DNA based on experimentally determined knotting probabilities. *Reactive and Functional Polymers* 131, 243–250. doi:https://doi.org/10.1016/j.reactfunctpolym.2018.08.002

Savelyev, A. (2012). Do monovalent mobile ions affect DNA's flexibility at high salt content? *Phys. Chem. Chem. Phys.* 14, 2250–2254. doi:10.1039/C2CP23499H

Sobel, E. S. and Harpst, J. A. (1991). Effects of Na+ on the persistence length and excluded volume of T7 bacteriophage DNA. *Biopolymers* 31, 1559–1564. doi:https://doi.org/10.1002/bip.360311311

Virnau, P., Rieger, F. C., and Reith, D. (2013). Influence of chain stiffness on knottedness in single polymers. *Biochem. Soc. Trans.* 41, 528–532
